# Activity and Thermal Aging Stability of La_1−x_Sr_x_MnO_3_ (x = 0.0, 0.3, 0.5, 0.7) and Ir/La_1−x_Sr_x_MnO_3_ Catalysts for CO Oxidation with Excess O_2_

**DOI:** 10.3390/nano13040663

**Published:** 2023-02-08

**Authors:** Catherine Drosou, Ersi Nikolaraki, Vasilios Nikolaou, Evangelia Koilia, Georgios Artemakis, Antonios Stratakis, Antigoni Evdou, Nikolaos D. Charisiou, Maria A. Goula, Vasilios Zaspalis, Ioannis V. Yentekakis

**Affiliations:** 1 Laboratory of Physical Chemistry & Chemical Processes, School of Chemical and Environmental Engineering, Technical University of Crete, 73100 Chania, Crete, Greece; 2 School of Mineral Resources Engineering, Technical University of Crete, 73100 Chania, Crete, Greece; 3 Department of Chemical Engineering, Aristotle University of Thessaloniki, 54124 Thessaloniki, Greece; 4 Chemical Process & Energy Resources Institute/Center for Research & Technology Hellas (CPERI/CERTH), 6th km Harilaou-Thermis, Thermi, 57001 Thessaloniki, Greece; 5 Department of Chemical Engineering, University of Western Macedonia, 50100 Koila, Kozani, Greece

**Keywords:** CO oxidation, excess O_2_ conditions, LSM perovskites, iridium nanoparticles, hysteresis phenomena, isothermal steady-state multiplicity

## Abstract

The catalytic oxidation of CO is probably the most investigated reaction in the literature, for decades, because of its extended environmental and fundamental importance. In this paper, the oxidation of CO on La_1−x_Sr_x_MnO_3_ perovskites (LSMx), either unloaded or loaded with dispersed Ir nanoparticles (Ir/LSMx), was studied in the temperature range 100–450 °C under excess O_2_ conditions (1% CO + 5% O_2_). The perovskites, of the type La_1−x_Sr_x_MnO_3_ (x = 0.0, 0.3, 0.5 and 0.7), were prepared by the coprecipitation method. The physicochemical and structural properties of both the LSMx and the homologous Ir/LSMx catalysts were evaluated by various techniques (XRD, N_2_ sorption–desorption by BET-BJH, H_2_-TPR and H_2_-Chem), in order to better understand the structure–activity–stability correlations. The effect of preoxidation/prereduction/aging of the catalysts on their activity and stability was also investigated. Results revealed that both LSMx and Ir/LSMx are effective for CO oxidation, with the latter being superior to the former. In both series of materials, increasing the substitution of La by Sr in the composition of the perovskite resulted to a gradual suppression of their CO oxidation activity when these were prereduced; the opposite was true for preoxidized samples. Inverse hysteresis phenomena in activity were observed during heating/cooling cycles on the prereduced Ir/LSMx catalysts with the loop amplitude narrowing with increasing Sr-content in LSMx. Oxidative thermal sintering experiments at high temperatures revealed excellent antisintering behavior of Ir nanoparticles supported on LSMx, resulting from perovskite’s favorable antisintering properties of high oxygen storage capacity and surface oxygen vacancies.

## 1. Introduction

The catalytic oxidation of CO has a wide range of applications. Among them, the most important are those for cars’ emission control, room-temperature CO oxidation and the selective removal of CO from hydrocarbons reforming gas to produce pure H_2_ (preferential CO oxidation, PROX) [1,2,3,4,5,6,7,8,9,10]. In addition, as a reaction with the absence of by-products, it is often used as a probe system for evaluating a wide variety of innovative catalysts [11,12]. However, at the same time, the catalytic oxidation of CO is a perfect example of a very complex reaction in terms of mechanistic behavior, which depending on the conditions can exhibit steady-state multiplicity, oscillatory or even chaotic behavior [1,8,13,14,15,16,17,18,19,20]. All the above rank this reaction as one of the most studied in heterogeneous catalysis, which continues with undiminished interest even today.

Perovskites, materials under the general formula ABO_3_, are characterized by remarkable thermal stability and mobility of oxygen ions while exhibiting good catalytic activity in redox reactions, typically at elevated temperatures [21,22,23]. The partial replacement of the A and/or B sites of a perovskite by other cations, with the same or different valences, results in so-called substituted A_1−y_A’yB_1−x_B’_x_O_3±δ_ perovskites, with modified bulk, surface and redox properties, particularly those of oxygen ion storage capacity (OSC) and mobility, as well as surface oxygen vacancy population, which are key properties in the catalysis of both oxides and supported on oxides metal catalysts [23,24,25,26,27,28]. The important role of these properties in the catalytic behavior of materials was, for example, recently shown in Refs. [24,25,26,27,28]. Specifically, Zhang et al. [24], using a “mechano-chemical” strategy involving Na-assisted milling of a NiCo_2_O_4_ spinel catalyst, enabled the production of a multidefective spinel catalyst with effectively enhanced surface defects (oxygen vacancies) and oxygen ion mobility, and thus a material significantly outperforming pristine NiCo_2_O_4_ and Co_3_O_4_ catalysts in propane oxidation [24]. Yu et al. [25] used a similar method, combining ball milling and selective atom removal, to obtain a high-performance CH_4_ combustion γ-MnO_2_ catalyst starting from a LaMnO_3_ perovskite. The superior catalytic performance was attributed to the increased bulk and surface oxygen defects, Mn^4+^/Mn^3+^ and O_surf_/O_latt_ ratios, reducibility and specific surface area of γ-MnO_2_ compared to LaMnO_3_ [25]. A similar corrosion-type procedure, described as selective dissolution, was proposed by Si et al. [26] as an efficient method for the selective removal of A cations from ABO_3_ perovskite structures, thus providing highly effective redox catalysts; this because the surface of the typically low-specific-surface-area perovskite materials is preferentially occupied by the catalytical inactive A-cations. Using a LaMnO_3_ perovskite and dilute HNO_3_ as a leaching agent, La was selectively removed from the perovskite structure to provide a γ-MnO_2_-like material with significantly higher CO oxidation activity compared to LaMnO_3_, due to its larger surface area and reducibility at low temperatures, and mainly due to its higher population of surface oxygen species [26]. Surface defects (oxygen vacancies) and oxygen storage capacity and mobility of CeO_2_-based mixed oxide supports used for the dispersion of Rh and Ir nanoparticles on their surface were found to be key factors in inducing a bifunctional mechanism, as well as in determining the oxidation state and local surface chemistry of the nanoparticles, thereby promoting the dry reforming of methane (DRM) reaction [27,28].

In any case, perovskite materials, either used as such or loaded with metal particles, permit the appropriate adjustment of the mentioned—key in catalysis—properties for improving their catalytic performance. Apart from the aforementioned modern methods, the conventional method based on the substitution of A and B sites with other cations is still widely applied, leading to advanced catalysts for various environmental and energy applications [23].

Perovskites based on the combination of La and Mn in the A and B positions, respectively, are among the most popular materials in the family. The partial substitution of La^3+^ by Sr^2+^ in the perovskite structure, i.e., La_1−x_Sr_x_MnO_3_, can enhance the redox properties of the material by increasing the oxidation states of the B cation (Mn^3+^) and consequently the material’s ability to generate oxygen vacancies, therefore introducing significant changes in their catalytic performance and their thermal stability [29]. Taking advantage of the aforementioned properties, perovskites could potentially also be used as “active” carriers for the deposition of metal nanoparticles, contributing to further enhancing the catalytic behavior of the latter [23].

Iridium is a relatively inexpensive noble metal compared to other platinum group metals (PGMs), with excellent properties in CO and VOCs oxidation reactions [30,31,32,33] and methane partial oxidation reactions [28,34,35], as well as NO_x_ and N_2_O reduction reactions [36,37,38,39]. However, the main inhibiting factor for Ir use as a catalyst in such practical applications is its high aggregation propensity under oxidizing environments and elevated temperatures [28,35,37,38]. Nevertheless, we have recently reported an efficient method to stabilize Ir nanoparticles, which is based on the use of supports with a high lattice oxygen storage capacity (OSC) and O^2−^ ion mobility values [40,41]. Perovskites as catalytic supports that possess these characteristics are potential candidate materials that could impart resistance properties to the sintering of noble or other metal nanoparticles dispersed on their surface. Among others, such materials are promising as oxygen carriers in chemical looping processes that have recently been of intense technological interest in natural gas and biogas processing [42,43,44]. 

Following the pioneering work of Nishihata et al. [45] that demonstrated the effectiveness and feasibility of using perovskite-based materials in automotive emission control technology, the interest in studying perovskites in three-way catalytic chemistry has been renewed [46]. However, among the main car pollutants and all fossil fuel combustion processes, CO dominates. Nevertheless, to the best of our knowledge, publications on the oxidation of CO over perovskite-type materials are still rare.

In the present work, four perovskites of the LSM family were prepared, namely La_1−x_Sr_x_MnO_3_ (x = 0.0, 0.3, 0.5 and 0.7), hereafter denoted as LSMx, with x indicating the Sr content of the material. These were subsequently used as supports for the dispersion of Ir nanoparticles, and both groups of materials (LSMx and Ir/LSMx) were studied for the CO oxidation reaction under O_2_ excess conditions. The physicochemical and structural properties of both LSMx and Ir/LSMx materials were evaluated by various techniques, such as X-ray diffraction (XRD), BET-BJH analysis of N_2_ adsorption–desorption isotherms, temperature programmed reduction (H_2_-TPR) and isothermal H_2_-chemisorption. The catalytic activity of the materials was comparatively evaluated under CO oxidation with excess O_2_ in their prereduced and preoxidized states, and before and after they were imposed to harsh oxidative thermal aging conditions (i.e., under conditions in which iridium nanoparticles are known to be particularly prone to aggregation) to obtain a thorough overview of their catalytic activity, stability and sintering performance.

## 2. Materials and Methods

### 2.1. LSMx and Ir/LSMx Synthesis

The perovskite materials La_1−x_Sr_x_MnO_3_ were prepared by the coprecipitation method [47]; their chemical composition denoted through LSMx coding, where x = 00, 30, 50 and 70 is used to express the % replacement of La by Sr in the perovskite formula (Table 1). Nitrate salts La(NO_3_)_3_∙6H_2_O (VWR Chemicals, 99.9%), Sr(NO_3_)_2_ (Sigma Aldrich, 99.0%) and Mn(NO_3_)_2_∙6H_2_O (Panreac, 98.0%) were used as metal precursors. An aqueous solution of the appropriate amounts of the nitrate salts was added to the precipitating agent (NaOH, VWR Chemicals, Radnor, PA, USA, 98.9%) solution. Following this, the precipitate product was filtered, washed, dried, deagglomerated and finally heated to 1000 °C for 6 h in air in order for the final perovskite structure to be obtained [42,43]. 

The deposition of Ir on the LSMx perovskites was achieved by impregnating the LSMx in an aqueous solution of IrCl_3_ (2 mg Ir/mL), with the appropriate amount of iridium in order to achieve a nominal loading of 2 wt% Ir in the final Ir/LSMx catalysts. The obtained catalyst precursors were dried at 110 °C for 12 h and then subjected to a reduction process (in 25% H_2_/He flow of 50 mL/min at 400 °C for 3 h) in order to remove residual chlorine and to avoid the formation of large Ir crystallites Ir [28,38]. Four Ir/LSMx catalysts were prepared following the above method (Table 1).

### 2.2. Catalysts Characterization Methods 

The textural characteristics of the LSMx and the corresponding 2 wt% Ir/LSMx perovskite-type catalysts were determined from N_2_ sorption–desorption isotherms at relative pressures in the range of 0.05–0.30 and a temperature of −196 °C performed on a Nova 2200e Quantochrome instrument. A mass of 150 mg of the material was placed in the instrument holder and degassed under vacuum for 12 h at 350 °C prior to measurements. The Brunauer–Emmett–Teller (BET) method was used to determine the total surface area (S_BET_) of the material, while the Barrett–Joyner–Halenda (BJH) model was applied to determine the pore volume and average pore size diameter.

The determination of the crystal structure of the materials was carried out by X-ray diffraction (XRD) measurements, on a BrukerAXS D8 Advance diffractometer at 35 kV and 35 mA with Cu Kα radiation and LynxEye detector with Ni-filter. The measurements were carried out in the 2θ angle range of 4–70 degrees with a scanning speed of 0.5 degrees per minute. Samples were calcinated in air at 400 °C for 1 h before XRD measurements. Crystallography Open Database (COD) was used for the identification of the crystal structures. The quantification of the phases in the samples was performed with the Rietveld method using BrukerAXS Topas software (COD, Crystallography Open Database). 

Isothermal (at 25 °C) hydrogen chemisorption (H_2_-chemisorption) experiments, as well as temperature-programmed reduction (H_2_-TPR) experiments in the temperature range 25–750 °C, were performed on a Quantachrome/ChemBet Pulsar TPR/TPD instrument equipped with an Omnistar/Pfeiffer Vacuum mass spectrometer as follows: 

For H_2_-chemisorption measurements, a mass of ~150 mg of the material was loaded on the quartz U tube holder of the instrument and pretreated with a 5% H_2_/He mixture (15 mL/min) at 550 °C for 1 h, followed by flushing with N_2_ (15 mL/min) at the same temperature for 30 min and cooling to room temperature under N_2_ flow. Then, pulses of pure hydrogen (280 µL) were injected until saturation, and the total hydrogen uptake per gram of sample (chemisorbed H_2_, V_Chem. H2_) was measured. These values were then used to calculate the hydrogen-to-metal ratio, H/Ir (i.e., the dispersion, D_Ir_) and the mean Ir crystallite size (d_Ir_) using Equations (1) and (2) [28]:

(1) $$D_{\mathrm{Ir}}=\frac{V_{\mathrm{Chem}.H2}\cdot F_{s}\cdot A_{\mathrm{Ir}}}{V_{\mathrm{mol}}\cdot X_{\mathrm{Ir}}}$$

(2) $$d_{\mathrm{Ir}}=\frac{6\cdot A_{\mathrm{Ir}}\cdot 10^{20}}{D_{\mathrm{Ir}}\cdot \rho_{\mathrm{Ir}}\cdot \alpha_{\mathrm{Ir}}\cdot N_{\mathrm{AV}}}$$

where V_Chem.H2_ (mL/g) is the H_2_ uptake in the chemisorption experiments, F_s_ is the hydrogen-to-metal correlation factor (equal to 2, assuming one-to-one correlation of adsorbed H atoms with metal sites, i.e., H−Ir), A_Ir_ is the atomic weight of Iridium (192.22 g/mol), V_mol_ (= 24,450 mL/mol) is the molar volume of an ideal gas at room temperature and 1 atm pressure, X_Ir_ is the iridium content of the catalyst (g_Ir_/g_cat_), ρ_Ir_ is the Ir metal density (22.5 g/mL), α_Ir_ is the cross-section area of Ir atom (0.12 nm^2^/atom), N_AV_ = 6.023 × 10^23^ molecules/mol is the Avogadro number and 10^20^ is a unit conversion factor when the units of parameters in Equations (1) and (2) are used as indicated above.

Then, using the same arrangement, the H_2_-TPR method was followed to obtain the reducibility profiles and determine the total oxygen storage capacity (t-OSC) of the materials (LSMx supports or the corresponding Ir/LSMx catalysts). For this purpose, the samples were first oxidized in situ at 750 °C for 30 min with a flow of 20% O_2_/He followed by cooling to 25 °C under the same flow. After purging the line by He flow for 10 min, the TPR experiment was conducted using a 15 mL/min flow of 1% H_2_/He and a heating rate of 10 °C/min up to ~750 °C. The H_2_-TPR profiles showing the reducibility characteristics of the materials and the total area of the H_2_ consumption peaks versus time were then used to determine the oxygen storage capacity (OSC) of the materials [27,28,37,41].

### 2.3. Catalytic Activity and Durability Evaluation Experiments

The catalytic activity and thermal stability experiments were performed in a quartz tubular (i.d. 3.0 mm) continuous flow fixed bed reactor typically loaded with 20 mg mass of catalyst (m_cat_ = 20 mg). Catalytic performance evaluation experiments on CO oxidation reaction were then conducted under conditions of excess O_2_ (1% CO + 5% O_2_, balance He at 1 bar), with a total flowrate of F_T_ = 160 mL/min, corresponding to a weight-basis gas hourly space velocity (wGHSV) = 480,000 mL/g∙h, in the temperature range of 50–400 °C, by increasing the temperature stepwise (~20–50 °C/step), remaining at each step for ~20 min for steady-state operation and keeping reactor feed conditions constant (light-off performance).

In order to examine the effect of catalyst state (i.e., prereduced or preoxidized) on its CO oxidation performance, all catalysts were subjected to two different pretreatment protocols before light-off experiments. Protocol #1: prereduction under a 25% H_2_/He flow (50 mL/min) at 400 °C for 1 h; Protocol #2: preoxidation under a 20% O_2_/He flow (50 mL/min) at 400 °C for 1 h.

The stability of the catalysts subjected to oxidative thermal aging conditions was studied by applying two successive aging steps, which included (I) in situ oxidative aging for 5 h at 600 °C, followed by (II) in situ oxidative aging for an additional 5 h at 750 °C; in both steps, a 20% O_2_/He flow was applied. After each aging stage (Ι and ΙΙ), the catalysts were subjected to a short reduction (with a flow of 25% H_2_/He for 30 min) at the corresponding aging temperature, then cooled at 150 °C for obtaining their light-off performance, followed by ca. 5 h operation at a constant temperature (T = 350 °C) for further performance evaluation. Feed conditions for these measurements were always the same (1% CO + 5% O_2_, wGHSV = 480,000 mL/g∙h). 

The analysis of reactants and products was carried out by on-line gas chromatography (Shimadzu 14 B with TC detector, He carrier gas and Porapak-N column). The conversion of CO (*X_CO_*) was calculated by using Equation (3):

(3) $$X_{CO}\left(\%\right)=100\frac{F_{in}{\left[CO\right]}_{in}-F_{out}{\left[CO\right]}_{out}}{F_{in}{\left[CO\right]}_{in}}$$

where *F_in_* and *F_out_* are the total gas flowrate (mL/min) in the inlet and outlet of the reactor, respectively, and [*CO*]*_in_* and [*CO*]*_out_* the *v*/*v* fraction of CO in the reactor inlet and outlet gas stream. 

## 3. Results and Discussion

### 3.1. Textural, Structural and Physicochemmical Properties of the Matarials

The textural characteristics of LSMx and Ir/LSMx are summarized in Table 1. The total surface area (S_BET_) of the LSMx perovskites ranges from 6.8 to 12.0 m^2^/g. The addition of 2 wt% Ir (Ir/LSMx materials) caused only a slight decrease in surface area, indicating negligible pore blocking by the formed Ir particles. This suggests the formation of very small iridium nanoparticles through the modified wet impregnation method applied. This is consistent with the average Ir particle size estimated from the H_2_-chemisoprtion experiments using Equations (1) and (2), which show particle sizes in the range of 1.0–1.2 nm and high dispersions of ca. 61–73% (Table 1).

**Figure 1:**
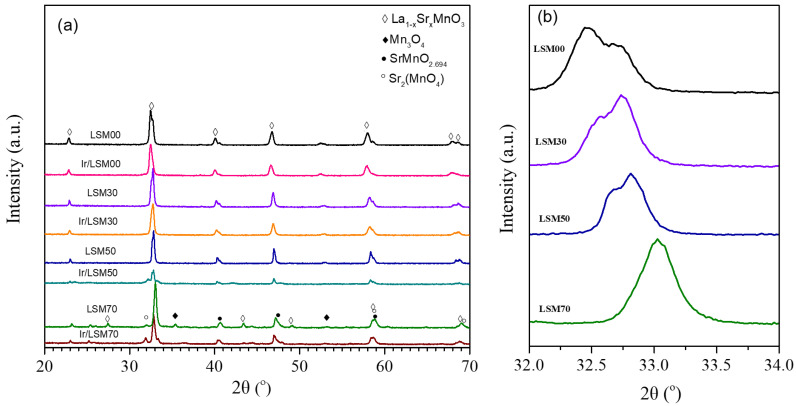
(**a**) XRD patterns of LSMx and 0.2 wt% Ir/LSMx catalysts, for 20° < 2θ < 70°; (**b**): magnification of the LSMx diffractograms in the region 32° < 2θ < 34°.

Structural analysis of all materials was carried out by XRD. The diffractograms of the samples are shown in Figure 1a, where the formation of the lanthanum manganate perovskite structure is confirmed with the main peak located at an angle, 2θ, between 32.4 and 33.1. As the Sr content increases, the peak shifts to larger angles (Figure 1b), which means that the unit cell is being contracted. This result is in agreement with the literature and provides a strong indication that charge compensation of the large substitutional Sr ions in La positions (i.e., according to the Kröger–Vink notation [48], noted as $Sr_{La}^{\prime }$) does not proceed through the creation of oxygen vacancies ($V_{O}^{\cdot \cdot }$) that would have resulted in a unit cell expansion, but through the oxidation of trivalent Mn to tetravalent ($Mn_{Mn}^{•}$) [29,44].

In addition, a splitting tendency of the peak is observed (Figure 1b), which, however, tends to disappear as the substitution of La by Sr increases. This is possibly related to the gradual transformation of the orthorhombic perovskite structure to cubic. Moreover, for high Sr contents (i.e., x = 0.7) the presence of other crystalline structures such as oxides or mixed oxides of Mn and La are observed, while due to its high dispersion (i.e., nanoparticles of the order of ~1 nm; Table 1) no IrO_2_ was detected.

**Figure 2:**
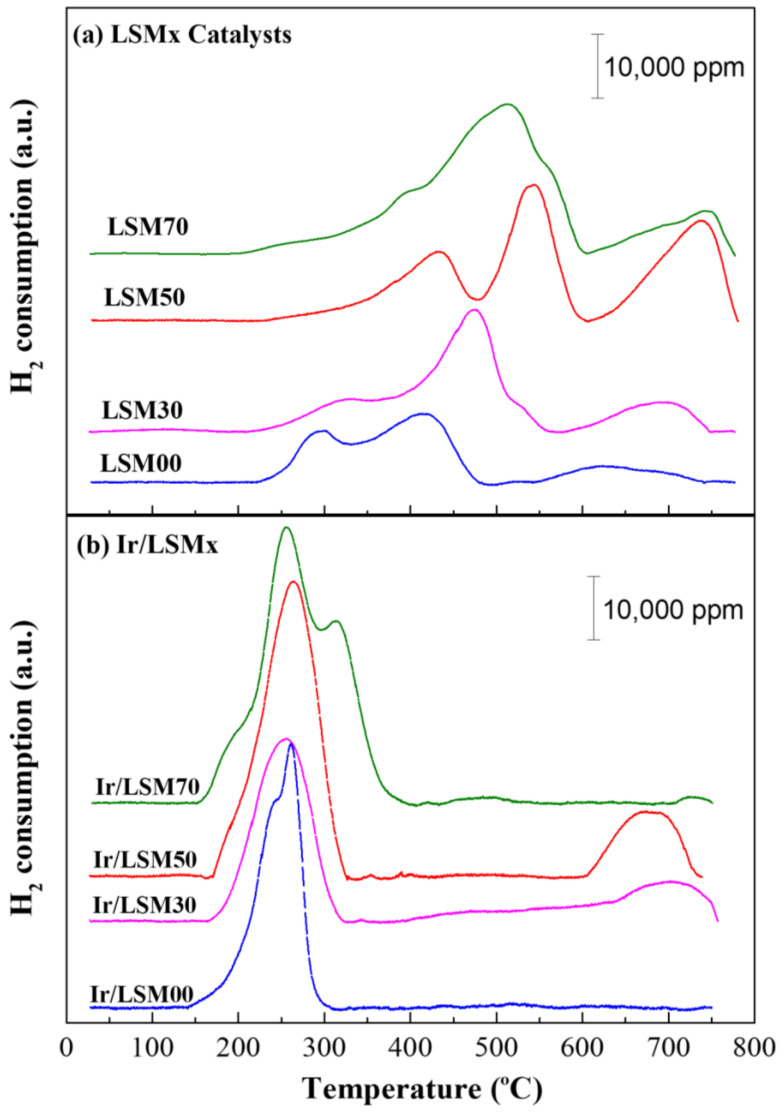
H_2_-TPR profiles (H_2_ consumption versus temperature) of LSMx (**a**), and 2 wt% Ir/LSMx (**b**) catalysts.

The profiles of H_2_ consumption versus temperature for the LSMx and 2 wt% Ir/LSMx catalysts, obtained from the H_2_-TPR experiments, are depicted in Figure 2a,b, respectively. Determining the total amount of H_2_ consumed from the integrated area of the respective TPR profiles in the time interval of the experiment, the total oxygen storage capacity (t-OSC) of the samples can be estimated [27,28,37]. All samples show high t-OSC values, ranging from 670 to 1350 μmol O_2_/g, which progressively increase as the substitution of La by Sr increases (Table 1). Actually, these values represent the total of labile lattice oxygen up to 750 °C, which concerns the redox couples Mn^4+^/M^3+^ (typically at T < 500 °C), Mn^3+^/Mn^2+^ (at T > 500 °C), and Ir^4+^/Ir^0^ if present (at T < ~300 °C) (Figure 2). As stated during the discussion of the XRD results, the incorporation of substitutional Sr^2+^ into the lattice increases the Mn^4+^ content. Subsequently, the main reduction process (that actually determines the OSC) is the reduction of Mn^4+^ to Mn^3+^, which proceeds with simultaneous oxygen removal and the creation of oxygen vacancies [28]:

(4) $$O_{O}^{\left(2-\right)\times }+2Mn_{Mn}^{\left(4+\right)\cdot }\overset{}{\Leftrightarrow }2Mn_{Mn}^{\left(3+\right)\times }+V_{O}^{\cdot \cdot }+\frac{1}{2}O_{2}\left(g\right)$$

Thus, the higher the Sr^2+^ content, the higher the concentration of Mn^4+^, and consequently, the higher the amount of the evolved oxygen (OSC), which explains the experimentally observed results.

It is also evident from the results of Figure 2a that the most readily reducible perovskite of the group is LaMnO_3_ (LSM00), and that the reducibility of LSMx progressively shifts to higher temperatures as their Sr content increases. The Mn^4+^ reduction and vacancy creation defect equilibria constants (or equivalently the Mn^4+^ reduction enthalpies) have been found to depend quite strongly on the Sr content. In particular, they decrease with increasing Sr content [49]; more about the defect chemistry of La_1−x_Sr_x_MnO_3_ perovskites can also be found in Ref. [44]. This may provide an explanation for the gradual shift of homologous peaks to higher temperatures with increasing Sr content. However, the results in Figure 2b show that addition of Ir shifts the reducibility of all perovskites to much lower temperatures, in the region ca. 200–400 °C. This well-known phenomenon for supported PGM nanoparticles is due to an enhanced spillover of hydrogen atoms from the Ir particles to the LSMx support favoring the reducibility of the latter; in the absence of Ir nanoparticles, this process is limited by H_2_ dissociation [40,50].

### 3.2. Catalytic Activity Evaluation on CO Oxidation

#### 3.2.1. Light-Off Performance of Prereduced and Preoxidized Catalysts

**Figure 3:**
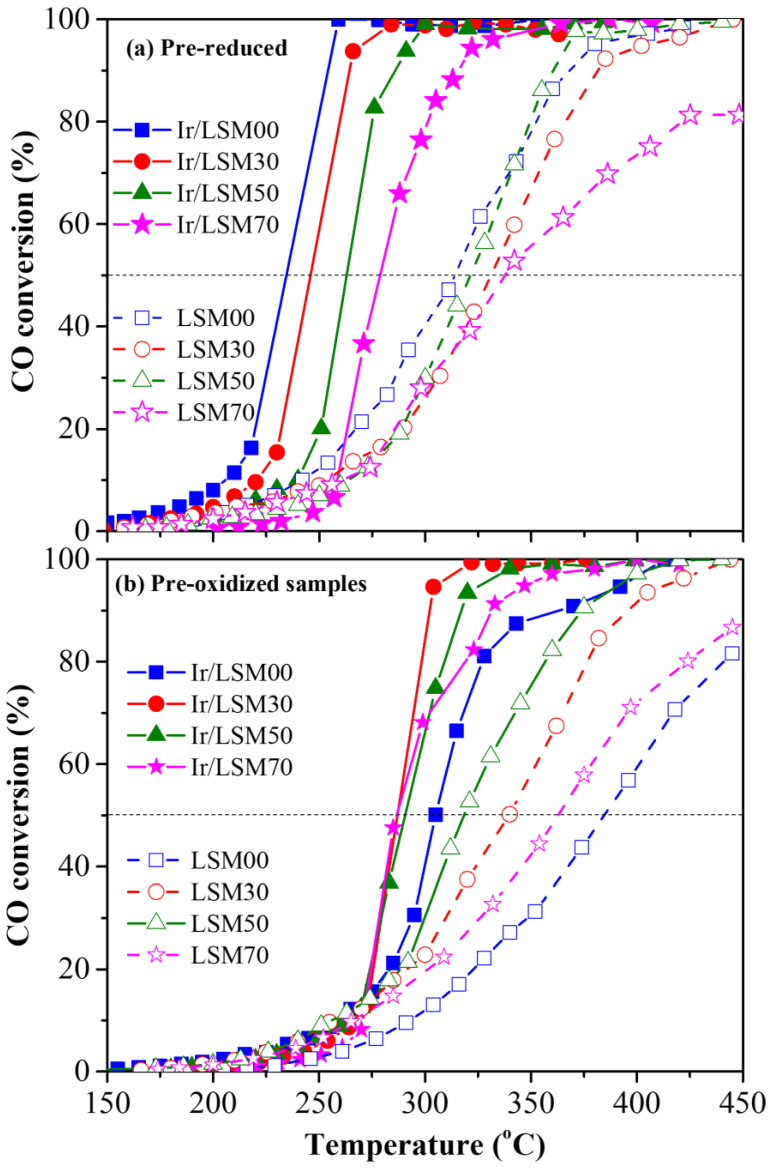
Conversion of CO as a function of temperature (light-off diagrams), for prereduced (**a**) and preoxidized (**b**) LSMx and Ir/LSMx catalysts. Experimental conditions: 1.0% CO, 5.0% O_2_, He balance at 1 bar, F_T_ = 160 mL/min, catalyst mass m_cat_ = 20 mg, wGHSV = 480,000 mL/g∙h. Open symbols and dashed lines → LSMx; solid symbols and solid lines → Ir/LSMx).

The catalytic performance of both bare LSMx and Ir/LSM materials was studied in terms of two pretreatment protocols: Protocol #1 referring to prereduced materials and Protocol #2 referring to preoxidized ones. The results are depicted in the light-off diagrams (CO conversion versus Temperature) of Figure 3. Specifically, Figure 3a shows the light-off performance of the prereduced LSMx and Ir/LSMx catalysts (Protocol #1), while Figure 3b shows those of the preoxidized ones (Protocol #2).

Both the bare LSMx and the iridium-enriched Ir/LSMx perovskites, regardless of their pretreatment conditions, show appreciable activity in terms of the CO oxidation reaction, with their CO conversion activity being ignited at ca. 150 °C (Figure 3). However, for both pretreatments, the iridium-containing materials (Ir/LSMx) clearly outperform the corresponding bare LSMx perovskites (Figure 3). The best-performing catalyst appears to be Ir/LSM00 (Ir/LaMnO_3_) in its prereduced state, achieving 100% CO conversion as low as 258 °C, while the worst-performing is LSM00 (LaMnO_3_) in its preoxidized state, achieving only ~81% CO conversion at the highest temperature investigated (450 °C).

**Figure 4:**
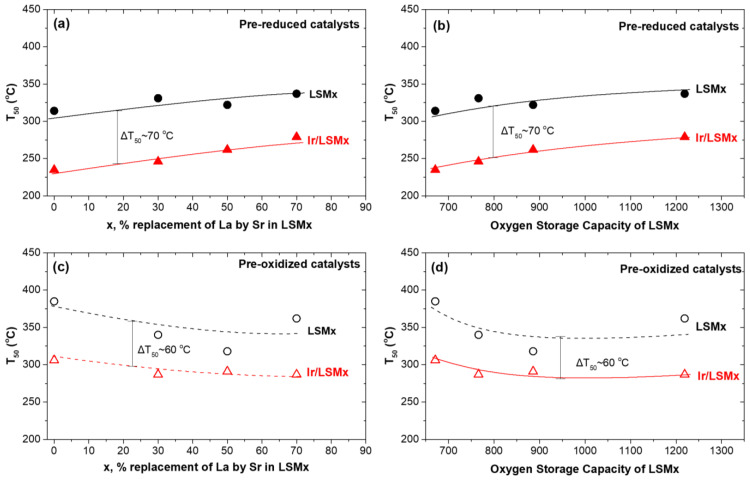
The temperature for 50% conversion of CO (T_50_) achieved by LSMx (black, circles) and Ir/LSMx (red, triangles) catalysts in their prereduced (**a**,**b**) and preoxidized (**c**,**d**) states as a function of the % replacement of La by Sr (**a**,**c**) and OSC of the LSMx (**b**,**d**).

For the sake of an easier overview of the changes in the activity of LSMx and Ir/LSMx catalysts as a function of the pretreatment that they underwent and their composition, Figure 4 was plotted, which depicts the temperature for 50% CO conversion (T_50_) as a function of perovskite composition (x, % replacement of La by Sr) and their corresponding OSC values for both imposed pretreatments (prereduced and preoxidized). Specifically, Figure 4a,b depict T_50_ of LSMx and Ir/LSMx as a function of x or OSC, respectively, for prereduced catalysts, while Figure 4c,d depict the analogous behaviors for the preoxidized materials. The following main observations and relevant discussion can be made:

An almost constant superiority of Ir/LSMx catalysts over LSMx, yielding a difference of about ΔT_50_ = 70 °C and ΔT_50_ = 60 °C in the prereduced and preoxidized states of the catalysts, respectively, is recorded through the almost parallel changes in T_50_ of LSMx and Ir/LSMx as a function of perovskite composition (x) and OSC for both pretreatments (prereduction and preoxidation) imposed. This implies that Ir nanoparticles’ CO oxidation activity constantly outperforms that of the bare LSMx perovskites throughout the whole range of compositions and OSCs of the latter. In addition, this result probably reflects a rather independent contribution of the Ir nanoparticles and the LSMx support to the overall activity of the Ir/LSMx material. That is, changes in the composition of LSMx undoubtedly cause some changes in its CO oxidation activity, but do not appear to cause any appreciable change in the activity of the Ir nanoparticles, with the result that the total activity of Ir/LSMx appears as the sum of the contributions of the two components (Ir and LSMx) of the Ir/LSMx material.

For the prereduced LSMx and Ir/LSMx catalysts, increasing the Sr content (and OSC value) caused a systematic degradation of their activity, i.e., higher T_50_ values (Figure 4a,b). However, the opposite is true for the preoxidized catalysts where T_50_ values decreased systematically upon increasing x and OSC of the perovskite (Figure 4c,d). Consistent with the reducibility characteristics of the LSMx perovskites (Figure 2a), this implies that the Mn^3+^ oxidation state of manganese most likely favors the CO oxidation reaction on the surface of the perovskite. This oxidation state of Mn in the perovskite structure increases the oxygen defects in its matrix, and consequently the bulk and surface oxygen vacancies. The latter are necessary for the adsorption of gas-phase CO and O_2_ to activate the CO oxidation reaction [51,52,53,54]. It is noted, however, that the mechanism of CO oxidation on perovskite surfaces is still not fully clear [51], and depending on the conditions and materials used, Langmuir–Hinshelwood, Eley–Rideal and Mars–van Krevelen mechanisms have been postulated [51,52,53,54,55]. Nonetheless, the role of surface oxygen vacancies is important for the activation of the gas-phase dioxygen, a process that involves its adsorption and dissociation, and thus refilling of the vacancies.

Considering the Mars–van Krevelen-type mechanism (participation of lattice oxygen) in perovskite-catalyzed CO oxidation, the observed activity order of LSM00 > LSM30 > LSM50 > LSM70 as a function of x (and OSC) for the prereduced LSMx perovskites can be rationalized in terms of their reducibility behavior found by H_2_-TPR experiments (Figure 2a); the easiness of reducibility of the LSMx perovskites follows the same order as their activity. Nevertheless, CO oxidation on LSMx was found to be activated in the temperature range of ca. 200–400 °C (Figure 3), where the reducibility of LSM00 exceeds that of the other perovskites of the group whose reducibility is shifted to higher temperatures (Figure 2a). This favors the Mars–van Krevelen mechanism on LSM00 at lower temperatures over the others, and thus its activity, as indeed observed (Figure 4a,b).

The opposite trend was observed for the preoxidized catalysts (Figure 4c,d), i.e., an increase in activity with increasing x, and therefore, the OSC of the material can be understood with similar considerations. The higher the OSC of the LSMx perovskite, the more prestored oxygen in its lattice during preoxidation, and thus the more availability of lattice oxygen to participate in the redox Mars–van Krevelen mechanism of the reaction, enhancing the rate.

#### 3.2.2. Heating (Light-Off)/Cooling (Light-Out) Cycles and Hysteresis Phenomena

**Figure 5:**
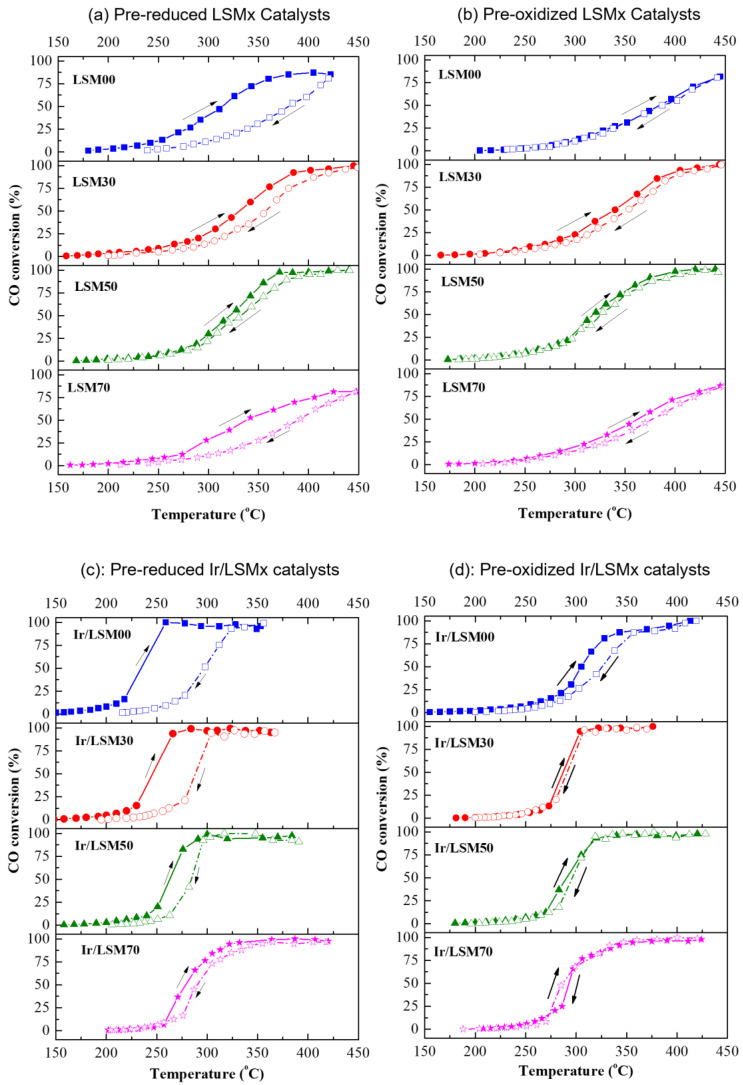
Light-off/light-out behavior of prereduced LSMx (**a**), preoxidized LSMx (**b**), prereduced Ir/LSMx (**c**) and preoxidized Ir/LSMx (**d**) catalysts and prereduced (**a**) and LSMx (**b**) catalysts. Display of inverse temperature (clockwise) hysteresis phenomena; arrows show the direction of the experiment.

The behavior of prereduced and preoxidized LSMx and Ir/LSMx catalysts during heating/cooling cycles under CO oxidation by excess O_2_ was also investigated, and the results are presented in Figure 5. As can be observed, by gradually increasing the temperature of the LSMx and Ir/LSMx catalysts from ~150 °C to ~450 °C (ignition/light-off path) and then cooling them down (extinction /light-out path), their activity exhibits isothermal steady-state multiplicity, i.e., CO conversion values at the same temperature do not coincide (Figure 5). A clockwise rate hysteresis, also referred to in the literature as “inverse” hysteresis [56], is therefore observed mainly over the prereduced LSMx (Figure 5a) and Ir/LSMx (Figure 5c) catalysts; the phenomenon is practically vanished over the preoxidized homologous catalysts (Figure 5b,d).

Such hysteresis phenomena are not uncommon in heterogeneous catalysis, especially in CO oxidation catalysis, where in many cases these are followed by isothermal self-sustained oscillations [1,13,14,15,16,17,18,19,20,57,58]. Among other considerations, the origin of the hysteresis and oscillatory phenomena can be attributed to changes in the oxidation state and/or restructuring of the active phase. For example, rate oscillations obtained during studies of CO oxidation on Pd(110) model catalyst [16] and Pt/γ-Al_2_O_3_ [17] were attributed to the reversible formation/decomposition of surface oxide phases of the metals. Furthermore, as solid electrolyte potentiometry (SEP)-aided studies of CO oxidation on polycrystalline Pt demonstrated that the experimentally observed isothermal rate bistabilities correspond to Pt^0^ (highly active phase) and PtO_2_ (less active phase) states of platinum and that the self-sustained rate oscillations that occurred were well constrained between these limits of catalytic activity, it was concluded that the origin of rate oscillations is the transient formation/decomposition of surface PtO_2_ [13,14]. The inverse hysteresis behavior of CO oxidation during CO/NO/O_2_ reaction over a monolithic diesel oxidation model catalyst (Umicore AG & Co. KG, Pt/Al_2_O_3_) was attributed to reversible oxidation of Pt [56]. *Operando* transmission electron microscopy-aided studies [57], showed that Pd nanoparticles exhibit periodic round-to-flat transitions altering their facets during CO oxidation reactions, which were considered responsible for the observed spontaneous rate oscillations appearing in both O_2_- and CO-rich reaction conditions, and as the authors claim, were unlikely to be caused by the oxidation of the surface, as argued in Ref. [58] for the oscillatory behavior of Pd-catalyzed CO oxidation investigated by means of *operando* X-ray diffraction experiments.

Regarding the inverse hysteresis observation in the present work, of particular interest are (i) the dependence of the hysteresis loop width on the perovskite composition (x, % replacement of La by Sr), which gradually compresses as x increases (Figure 5a,c); (ii) the hysteresis being less evident, practically vanished, over the preoxidized catalysts (Figure 5b,d); and (iii) the loop amplitude being more pronounced on the Ir-loaded materials (Ir/LSMx) compared to the unloaded ones (LSMx) (Figure 5a,c).

The dependence of the hysteresis on the perovskite composition (observation i) and the oxidation state of the catalyst (observation ii) enable us to postulate the origin of the phenomenon. Therefore, observations (i) and (iii) suggest that the hysteresis is related to both components (Ir and LSMx) of the catalyst, while observation (ii) indicates an unambiguous dependence on the oxidation state of the material.

Thus, starting, for example, the heating/cooling cycle experiment with a prereduced Ir/LSMx catalyst, the preimposed metallic state of the iridium nanoparticles (Ir^0^; more active phase than IrO_2_ in CO oxidation) together with the partially reduced manganese (Mn^3+^) in the support, drives the system to the highest possible CO conversion performance (consistent to the steady-state results of Figure 3a). As the system reaches high temperatures where 100% CO conversion is achieved, the catalyst now experiences a net oxidizing environment due to excess O_2_, which causes at least partial (surface) oxidation of the Ir nanoparticles as well as of the LSMx support towards Mn^4+^ state. Then, during the cooling (light-out) step of the hysteresis experiment, as expected, the catalyst operates at a lower catalytic activity that corresponds to the IrO_2_ and Mn^4+^ phases, which are less active in CO oxidation.

**Figure 6:**
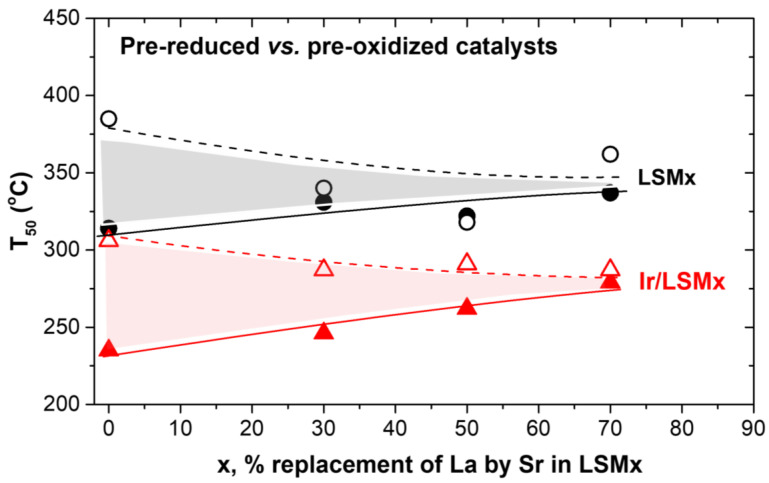
Activity limits in terms of T_50_ between prereduced (solid lines and symbols) and preoxidized (dashed lines and open symbols) LSMx (black lines and symbols) and Ir/LSMx (red lines and symbols) catalysts obtained from the light-off catalysts’ performance of Figure 3.

According to this suggestion, the upper and lower limits of the inverse hysteresis should be constrained in between the behaviors of the prereduced and preoxidized catalysts, respectively, as indeed appears to be the case for both LSMx and Ir/LSMx materials. Figure 6 was drawn from the results of the light-off performances of the prereduced and preoxidized LSMx and Ir/LSMx catalysts of Figure 3, confirming the above. As shown in Figure 6, the activity thresholds (ΔT_50_) between prereduced and preoxidized catalysts converge as x increases, in good agreement with the damping of the hysteresis loop width with respect to x observed in the heating/cooling experiments of Figure 5.

In the case of starting the heating/cooling cycle experiment with preoxidized catalysts, in line with the above considerations, the catalyst is in its oxidized state both during the heating (light-off) and during the cooling (light-out) steps. Therefore, in both temperature directions, the catalyst will work with the same activity, that which characterizes its less active oxidized state, and without hysteresis phenomena, as indeed confirmed by the results shown in Figure 5b,d.

### 3.3. Ir/LSMx Performance Stability after Oxidative Thermal Aging Treatments

The stability of performance of the Ir/LSMx catalysts on CO oxidation reaction, after their exposure to two consecutive oxidative thermal aging stages, was investigated, with the aim of determining whether the perovskites characterized by high values of oxygen storage capacity (OSC) and O^2−^ mobility are able, as supports, to stabilize the catalytic behavior of the Ir nanoparticles that are prone to agglomerating, as it was recently found to be the case with high OSCs CeO_2_-based supports [28,35,37,38,40,41].

**Figure 7:**
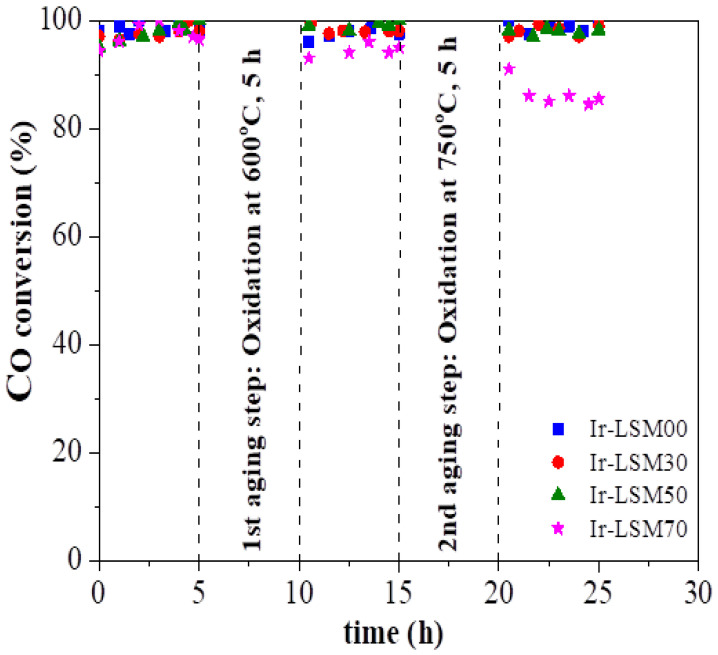
Control of the performance of the Ir/LSMx catalysts after each aging step, at 350 °C. Other experimental conditions are the same as in Figure 3.

As can be clearly seen in Figure 7, all catalysts show remarkable stability of their CO conversion efficiency after the aging steps imposed at 600 °C and 750 °C for 5 h at each step; the only catalyst showing a slight deactivation was that with the highest Sr content (Ir/LSM70).

This sufficient stabilization against oxidative thermal sintering of Ir NPs at temperatures as high as 750 °C is an important result for practical applications, considering that iridium in its oxidation state (IrO_2_) is one of the most sensitive elements of the noble metal series, with a high aggregation propensity even at temperatures as low as ~450 °C [37,59,60], and the fact that such high temperatures are commonly reached in practice, e.g., in automotive emissions control systems [2,7,23,46] and methane-reforming processes [3,27,28,34,35]. These results indicate the operation of antisintering mechanisms protecting the catalysts’ nanostructure and the sensitive IrO_2_ nanoparticles, most likely on the basis of the model recently demonstrated in a series of studies [28,37,38,40,41] evidencing that the high OSC values of the catalysts’ supports can sufficiently induce such stabilization phenomena; the LSMx perovskites studied herein indeed have high OSC values (Table 1).

**Figure 8:**
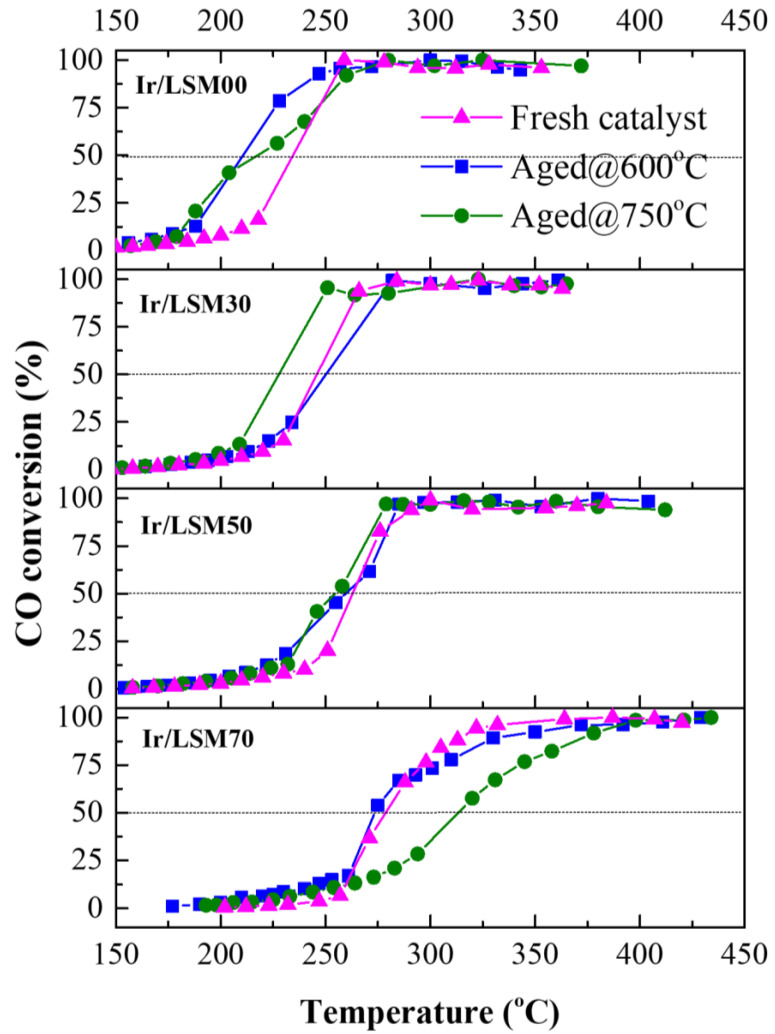
CO conversion profiles over fresh, Aged@600°C and Aged@750°C Ir/LSMx catalysts. Experimental conditions: 1.0% CO, 5.0% O_2_, He balance at 1 bar; FT = 160 mL/min, catalyst mass mcat = 20 mg, wGHSV = 480,000 mL/g∙h.

Figure 8 shows the complete behavior in light-off diagrams of the fresh and aged Ir/LSMx catalysts obtained immediately after the various pretreatments imposed in the thermal aging experiments, i.e., based on the time scale of Figure 7 at t = 0 h for the fresh catalysts, at t = 10 h for the Aged@600°C catalysts, and at t = 20 h for the Aged@750°C catalysts. It is noteworthy that the aged Ir/LSM00, Ir/LSM30 and Ir/LSM50 catalysts show a slightly better CO oxidation light-off behavior than their fresh counterparts. This is likely due to some slight redispersion of the Ir nanocrystallites during the oxidative thermal aging, as we recently observed to occur when the particles are dispersed in carriers that have a high population of surface oxygen vacancies and explained consistently via a model we developed [40,41]. According to this model, surface oxygen vacancies on catalyst supports can act as traps for atomic species detached from metal nanoparticles, eliminating their attachment from larger nanoparticles, leading to particles’ agglomeration via the Ostwald ripening (OR) mechanism. At the same time, the agglomeration of large nanoparticles via the particle migration and coalescence (PMC) mechanism is also eliminated though the intraparticle repulsive forces caused by the O^δ−^ layer created on the surface of nanoparticles [40,41]. Such effects, enabled by supports with high OSC and surface oxygen vacancies, appear to be at work herein as well; LSMx perovskites are materials that fully possess the necessary aforementioned prerequisites. The results of Figure 8 indicate sufficient stabilization of iridium nanoparticles supported on perovskite materials imposed at oxidative thermal aging treatment, or even a slight redispersion of them for all catalysts tested herein with the exception of Ir/LSM70, which shows a slight deactivation, especially after the second aging at 750 °C. It is suggested that due to the high Sr-content of the LSM70, other secondary phases besides the perovskite phase may exist in the mixed-oxide material. Therefore, a portion of Ir nanoparticles could be anchored on these, for which the requirements necessary to protect NPs from aggregation are not met.

**Figure 9:**
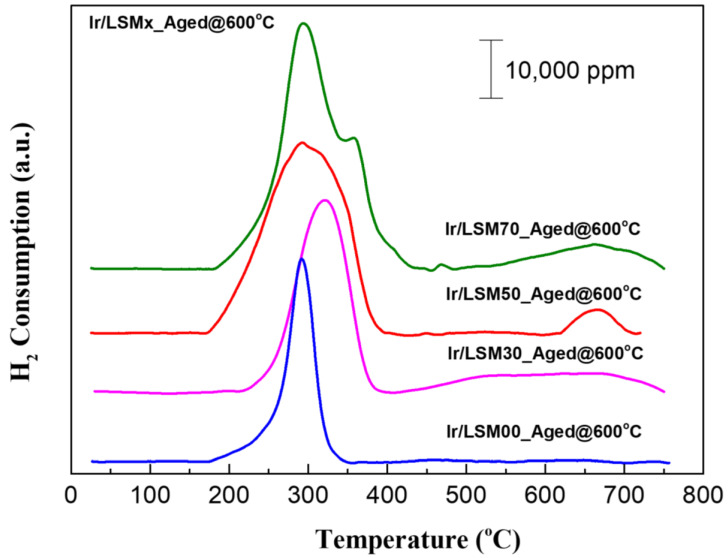
H_2_-TPR profiles of the Ir/LSMx catalysts thermally aged at 600 °C for 5 h in air.

Characterization experiments with BET, H_2_-TPR and H_2_-chemisorption techniques of Ir/LSMx catalysts after imposing oxidative thermal aging at 600 °C for 5 h further confirm the above conclusions. As shown in Figure 9, which depicts the H_2_-TPR profiles of the Aged@600°C for 5 h Ir/LSMx catalysts, no significant changes occurred in the conductivity characteristics of the catalysts after aging compared to their characteristics in their fresh state (Figure 2b)—this implies marginal textural and morphological modifications. Other textural and morphological characteristics of the aged catalysts included in Table 2 corroborate the same fact. Indeed, the total surface areas of the aged catalysts range between ~9–16 m^2^/g (Table 2), similar to those of their fresh counterparts (Table 1). The average Ir particle sizes of Ir/LSM00 and Ir/LSM30 remain unchanged at ca. 1.1 nm, a slight redispersion is recorded for Ir/LSM50 (from 1.0 nm → 0.9 nm), while on Ir/LSM70, the Ir NPs underwent slight sintering (from 1.2 nm → 1.5 nm); all observations are in good agreement with the results of Figure 8 and are consistent with the considerations made.

In summary, the present study identifies interesting phenomena that exist in the oxidation of CO by Ir loaded and unloaded lanthanum-strontium-manganate perovskite catalysts (LSMx and Ir/LSMx) in terms of their activity, steady-state multiplicity, and hysteresis phenomena, as well as their stability under intense oxidative thermal aging conditions as a function of their composition (x: % replacement of La by Sr). What is certain is that not all the observed phenomena are fully explained; however, interesting topics are opened for further investigation that are of both practical and fundamental importance.

## 4. Conclusions

The CO oxidation reaction under O_2_ excess conditions was investigated over LSMx (La_1−x_Sr_x_MnO_3_; x = 0, 0.3, 0.5 and 0.7) and Ir/LSMx perovskite-based materials. Both series of materials were active in the reaction, typically igniting at ca. 150–200 °C, with Ir/LSMx outperforming their LSMx counterparts offering T_50_ values (temperature for 50% CO conversion) ~70 °C lower.

The replacement of La by Sr in the perovskite formula impacted the catalytic performance of the materials, with the following main characteristics:

For prereduced materials, increasing x favored CO oxidation, while the opposite occurred for preoxidized materials, effects that are understood through the x-dependent reducibility, oxygen defects and OSC characteristics of the materials.

Changes in x were found to mainly affect the catalytic behavior of LSMx but not that of dispersed Ir nanoparticles, indicating that οn Ir/LSMx catalysts, the recorded catalytic activity is the sum of two independent contributions—that on Ir plus that on the support on which the reaction takes place—almost independently, without affecting each other.

Inverse hysteresis effects were observed, depending largely on both the pretreatment and the composition of the materials:

- The amplitude of hysteresis appears extended in prereduced LSMx and Ir/LSMx, while significantly limited in preoxidized ones, and also decreases with increasing x;
- The upper and lower limits of the hysteresis loop are bounded in between the behaviors of the prereduced and preoxidized catalysts.

These indicate that hysteresis is related to the Ir^0^ (up limit) and IrO_2_ (down limit) states of iridium, as well as to the Mn^3+^/Mn^4+^ oxidation states of manganese, with M^3+^ being more favorable for CO oxidation.

Oxidative thermal aging tests of Ir/LSMx catalysts at a temperature as high as 750 °C and for a prolonged time (10 h) demonstrated the excellent stability of Ir nanoparticles against sintering when supported on LSMx. This was attributed to the spontaneously created O^δ−^ layer on the surface of IrO_2_ nanoparticles from the O^2−^ species offered by the support, as well as the oxygen vacancies on the support surface, factors acting synergistically to prevent particle aggregation, in a manner recently described by our group [40,41]. Confirmation of the model through the present results, using another family of materials (perovskites) as a support, which possess the required properties, i.e., oxygen ion lability and mobility and surface defects, further supports its validity.

## Figures and Tables

**Table 1:** Textural and morphological characteristics and physicochemical properties of LSMx and 2 wt% Ir/LSMx catalysts.

Catalyst Code	Chemical Formula	S_BET_ (m^2^/g)	Mean Pores Diameter (nm)	Total OSC (μmol O_2_/g)	H_2_ Uptake V_Chem.H2_ (cm^3^/g)	Average Ir Particle Size (nm)	Ir Dispersion (H−Ir)
LSM00	LaMnO_3_	12.0	10.9	671	-	-	-
LSM30	La_0.7_Sr_0.3_MnO_3_	10.4	9.84	766	-	-	-
LSM50	La_0.5_Sr_0.5_MnO_3_	6.8	8.91	886	-	-	-
LSM70	La_0.3_Sr_0.7_MnO_3_	11.3	8.79	1219	-	-	-
Ir/LSM00	Ir/LaMnO_3_	9.7	11.9	753	0.80	1.1	0.63
Ir/LSM30	Ir/La_0.7_Sr_0.3_MnO_3_	10.5	9.96	981	0.79	1.1	0.62
Ir/LSM50	Ir/La_0.5_Sr_0.5_MnO_3_	6.2	8.11	1203	0.92	1.0	0.73
Ir/LSM70	Ir/La_0.3_Sr_0.7_MnO_3_	11.0	13.7	1348	0.78	1.2	0.61

**Table 2:** Total surface area (S_BET_) and Ir nanoparticles’ average size and dispersion of Ir/LSMx catalysts aged at 600 °C for 5 h in a 20% O_2_/He flow.

Catalyst Code	S_BET_ (m^2^/g)	H_2_ Uptake V_Chem.H2_ (mL/g)	Average Ir Particle Size (nm)	Ir Dispersion (H−Ir)
Ir/LSM00-Aged@600°C	14.1	0.82	1.1	0.64
Ir/LSM30-Aged@600°C	15.6	0.81	1.1	0.64
Ir/LSM50-Aged@600°C	9.1	0.99	0.9	0.78
Ir/LSM70-Aged@600°C	10.7	0.62	1.5	0.64

## Data Availability

The data is available at the laboratory/ies of origin upon request. www.pccplab.tuc.gr.
